# Advantages, Limitations and Recommendations for online learning during COVID-19 pandemic era

**DOI:** 10.12669/pjms.36.COVID19-S4.2785

**Published:** 2020-05

**Authors:** Khadijah Mukhtar, Kainat Javed, Mahwish Arooj, Ahsan Sethi

**Affiliations:** 1 Khadijah Mukhtar, BDS, MME. Assistant Professor, DME. University College of Medicine and Dentistry, The University of Lahore, Lahore, Pakistan; 2 Kainat Javed, MBBS, MME. Assistant Professor, DME. University College of Medicine and Dentistry, The University of Lahore, Lahore, Pakistan; 3 Mahwish Arooj, MBBS, M. Phil, MME, PhD Physiology. Associate Professor, Physiology and Director DME, University College of Medicine and Dentistry, The University of Lahore, Lahore, Pakistan; 4 Ahsan Sethi, BDS, MPH, MMEd, FHEA, MAcadMEd, FDTFEd, PhD Medical Education Assistant Professor, Institute of Health Professions Education and Research, Khyber Medical University, Peshawar, Pakistan

**Keywords:** COVID-19, Education, Medical, Undergraduate, Online learning

## Abstract

**Objective:**

During COVID-19 pandemic, the institutions in Pakistan have started online learning. This study explores the perception of teachers and students regarding its advantages, limitations and recommendations.

**Methods:**

This qualitative case study was conducted from March to April 2020. Using maximum variation sampling, 12 faculty members and 12 students from University College of Medicine and University College of Dentistry, Lahore were invited to participate. Four focus group interviews, two each with the faculty and students of medicine and dentistry were carried out. Data were transcribed verbatim and thematically analyzed using Atlas Ti.

**Results:**

The advantages included remote learning, comfort, accessibility, while the limitations involved inefficiency and difficulty in maintaining academic integrity. The recommendations were to train faculty on using online modalities and developing lesson plan with reduced cognitive load and increased interactivities.

**Conclusion:**

The current study supports the use of online learning in medical and dental institutes, considering its various advantages. Online learning modalities encourage student-centered learning and they are easily manageable during this lockdown situation.

## INTRODUCTION

The spread of COVID-19 has led to the closure of educational institutions all over the world. This tested the preparedness of universities to deal with a crisis that requires the help of advanced technology including hardware and software to enable effective online learning. Such closure accelerated the development of the online learning environments so that learning would not be disrupted.^1^ Many institutions have become interested in how to best deliver course content online, engage learners and conduct assessments. Hence, COVID-19 while being a hazard to humanity, has evolved institutions to invest in online learning.

Online learning systems are web-based software for distributing, tracking, and managing courses over the Internet.^2^ It involves the implementation of advancements in technology to direct, design and deliver the learning content, and to facilitate two-way communication between students and faculty.^3^ They contain features such as whiteboards, chat rooms, polls, quizzes, discussion forums and surveys that allow instructors and students to communicate online and share course content side by side. These can offer productive and convenient ways to achieve learning goals. In Pakistan, the institutions are using Microsoft Teams, Google meet, Edmodo and Moodle as learning management systems along with their applications for video conferencing.^4^ Other commonly used video conferencing solutions include Zoom, Skype for business, WebEx and Adobe connect etc.

According to our literature review, three previous studies were found,^5^-^7^ supporting online learning from Pakistan. The two studies at Dow University of Health Sciences, Karachi and Lahore Medical and Dental College, Lahore reported high student satisfaction with online learning modalities. The study from Khyber Pakhtunkhwa assessed the feasibility of online learning among students, trainees and faculty members. They reported good technology access, online skills, and preparedness for online discussions among participants across the medical education continuum.

With the increase in use of online modalities during COVID-19, it is necessary to assess their effectiveness with regards to teaching and learning from various stakeholders.^8^ Therefore, the current study explores the perception of faculty members and students regarding the advantages, limitations and recommendations for online learning in Pakistan. The study is timely as Higher Education Commission (HEC) is in the process of implementing online learning across all the universities in Pakistan. The findings will help identify the required changes on priority basis to make it more practical and worthwhile.

## METHODS

This qualitative case study was conducted from March to April 2020 in two medical and dental institutes. Ethical approval for this study was taken from ethical review board of University of Lahore (Ref No. ERC/02/20/02, dated February 25, 2020). Using maximum variation sampling 12 faculty members and 12 students from University College of Medicine and University College of Dentistry, Lahore were invited to participate. In addition to learning management system ‘Moodle’, these colleges have recently adopted ‘Zoom’ for interactive teaching in small and large group formats. The participants were also involved in online Problem-Based Learning sessions, along with regular online assessments during COVID-19 pandemic.

An interview guide was developed to explore faculty and students’ perception about online learning modalities, its advantages, limitations and recommendations. The interview guide was piloted to ensure comprehensiveness and then also validated by two medical education experts.^9^ Two interviewers who were not involved in teaching and assessment of students conducted four focus group interviews (n=6 in each group) with faculty members (n=12) and students (n=12) of medicine and dentistry. The faculty and students were from both basic sciences (1^st^ and 2^nd^ year) and clinical sciences (3^rd^, 4^th^ and final year). All interviews were recorded through ‘Zoom’ and subsequently transcribed verbatim. The data were thematically analyzed: compiling, disassembling, reassembling and interpretation by all the authors independently and then corroborated to ensure analytical triangulation.

## RESULTS

The faculty members were predominantly females from both basic and clinical sciences with age range from 30-64 years. The students were from all professional years of MBBS and BDS program (Table-I).

**Table-I:** Participant characteristics.

	Faculty (n=12)		Students (n=12)	
*Gender*				
Male	3(25%)		7(58%)	
Female	9(75%)		5(42%)	
*Age Group*				
18-29			12 (100%)	
30-49	9(75%)			
50-64	3(25%)			
	MBBS	BDS		
Basic Sciences	2 (34%)	3 (50%)		
Clinical Sciences	4 (66%)	3 (50%)		
			MBBS	BDS
1^st^ year			1	1
2^nd^ year			1	1
3^rd^ Year			1	2
4^th^ Year			1	2
5^th^ Year			2	

Total six themes, two each for advantages, limitations and recommendations were extracted from the transcribed data after qualitative analysis (Table-II).

**Table-II:** E-learning advantages, limitations and recommendations by Students and Faculty.

Themes	Sub-Themes	Excerpts

Advantages		
Flexibility	Remote learning	“It is useful in distant learning and during COVID 19 situation we can continue our education system”.
	Easy administration	“Our teacher has authority to unmute our mics and video. And can see and check whether we are listening attentively or not”.
	Accessibility	“The students who are not much confident, they contact through the WhatsApp easily”.
	Comfortable	“You can easily and comfortably listen to the lecture and learn”.
Student-centered learning	Self-directed learning	“I think eLearning is making good students more active and self-learner.”
	Asynchronous learning	“Second thing is that lectures have been recorded and will uploaded soon. It is easy for us to go back and go through the whole video for a summary or even revising it”.

*Limitations*		

Inefficiency	Unable to teach skills	“In anatomy, the study through models was good. But hands on training is not possible, the student will not be able to understand properly. Skills needs actual hands on training”.
	Lack of student feedback	“I find it annoying that during lectures you don’t have students feedback whether they are getting the point or not”.
	Limited attention span	“There is no continuity of lecture. We lose our concentration and the syllabus is so lengthy.”
	Lack of attentiveness	“As the students know that they will get the recordings, they don’t listen the lecture properly”.
	Resource intensive	“Lots of people might not be having these gadgets. Buying these gadgets comes an extra burden on them in such stressful situation”.
Maintaining academic integrity	Lack of discipline	“There is some problem coming with discipline, some students use to misbehave during lectures”.
	Plagiarism	As this system is new to everyone, it is difficult to have individual assessment. During assignment, they easily copy paste stuff from web.”

*Recommendations*		

Teaching and Assessment	Reduce cognitive load	“If you try to fix all the LOs in 40 minutes, then the interaction will not be possible.”
	Faculty development	“But we have to work with modality which institute has decided and using. But there is need of throughout training sessions”.
	Increase Interactivities	“We should interact with students who are not active listeners. The student interaction is only through the assessments and we will be able to access the students.”
	Incorporate CBL	“Case based learning is very important. It is the closest thing to the practical life. Making it easier, rather than making it complicated.”
	Revision classes	“After this lockdown when the university will open, there should be a revision session and practical work.”
	Integrate proper Assessment	“Assessment should be live videos and live recordings.”
	Develop SOP’s	“The student should log in through proper ID and only they can listen the lecture and see video”.
Quality enhancement	Proctoring	“There should be plagiarism software to check assignment.”
	Buy Premium Applications	“I guess institute should buy premium package for ZOOM app so there will no time limit while having lectures.”

### Advantages

Faculty opined that online learning helped ensure remote learning, it was manageable, and students could conveniently access teachers and teaching materials. It also reduced use of traveling resources and other expenses. It eased administrative tasks such as recording of lectures and marking attendance. Both the students and teachers had an opinion that online learning modalities had encouraged student-centeredness during this lockdown situation. The student had become self-directed learners and they learnt asynchronously at any time in a day.

### Limitations

Faculty members and students said that through online learning modalities they were unable to teach and learn practical and clinical work. They could only teach and assess knowledge component. Due to lack of immediate feedback, teachers were unable to assess students’ understanding during online lecturing. The students also reported limited attention span and resource intensive nature of online learning as a limitation. Some teachers also mentioned that during online study, students misbehaved and tried to access online resources during assessments.

### Recommendations

Teachers and students suggested continuous faculty development. They recommended a reduction in cognitive load and increased interactivities during online teaching. Those in clinical years suggested ways to start online Case Based Learning. However, some were also of the opinion that there should be revision classes along with psychomotor hands on teaching after the COVID-19 pandemic is under control. To enhance quality, they suggested buying premium software and other proctoring software to detect cheating and plagiarism.

## DISCUSSION

The current study reported advantages, limitations and recommendations to improve online learning during lockdown of institutions due to COVID-19 pandemic. This study interprets perspectives of medical/dental students and faculty members, which showed that online learning modalities are flexible and effective source of teaching and learning along with some pitfalls. According to the teachers and students, online learning is a flexible and effective source of teaching and learning as most of them agreed upon the fact that this helps in distant learning with easy administration and accessibility along with less use resource and time. Regardless of time limit, students can easily access the learning material. This flexibility over face to face teaching has been reported in the literature as well.^2^ The students also become self-directed learners, which is an important competency for encouraging lifelong learning among health professionals.^10^,^11^

Both the faculty members and students viewed inefficiency to teach psychomotor skills, resource intensiveness and mismanaged decorum during sessions as limitations of online learning. Even though, hands-on sessions such as laboratory and clinical skills teaching have been disrupted during COVID-19 pandemic, we believe that online simulated patients or role plays can be used teach history taking, clinical reasoning and communication skills. Sharing recorded videos of laboratory and clinical skills demonstration is also worthwhile. Faculty members also complained about lack of students’ feedback regarding understanding of subject. Research showed that regular two-way feedback helps enhance self-efficacy and motivation.^12^ The interaction between facilitator, learner and study material along with emotional and social support are essential ingredients for effective learning.^13^,^14^ Internet connectivity issues also adversely impacted learning through online modalities, however, simply improving internet package/speed would help resolve this. Government should also take immediate measures and telecommunication companies should invest in expanding its 4G services across the country.

Recommendations reflect that decorum can be maintained by thorough supervision of students, setting ground rules for online interaction, counselling and disciplinary actions.^15^ According to students, the attention span during online learning was even shorter than face to face sessions as also supported by the literature.^16^ This can be managed by using flipped classroom learning modalities, giving shorter lectures and increasing teacher-student interaction. As ‘assessment drives learning’, so online formative assessments can be conducted through Socrative and Kahoot etc. Faculty needs training and students orientation in using online learning tools.^17^ Investment in buying premium software packages will also help overcome many limitations and is therefore recommended.

### Limitations of the Study

As the study participants belonged to the medical and dental college from a single private-sector university of Punjab, therefore the findings are only applicable to similar contexts. For generalizability, a survey based on our findings should be conducted across the province or country. Despite the limitations, the findings offer an understanding of the advantages, limitations and recommendations for improvement in online learning, which is the need of the day.

## CONCLUSION

The current study supports the use of online learning in medical and dental institutes, considering its various advantages. E-learning modalities encourage student-centered learning and they are easily manageable during this lockdown situation. It is worth considering here that currently online learning is at a nascent stage in Pakistan. It started as ‘emergency remote learning’, and with further investments we can overcome any limitations. There is a need to train faculty on the use of online modalities and developing lesson plan with reduced cognitive load and increased interactivities.

### Author’s Contribution

**AS and MA** conceived the idea , designed the study and are responsible for integrity of research.

**KM and KJ** collected the data.

All the authors contributed towards data analysis and writing the manuscript and approved the final version.
